# Clinical Lectures on Surgical Cases in the Buffalo Hospital of the Sisters of Charity

**Published:** 1870-11

**Authors:** J. F. Miner


ART. III.—*Clinical Lectures on Surgical Cases in the Buffalo Hospital of the Sisters of Charity*, by Prof. J. F. MINER, M. D., *reported by W. W. Miner, member of the Class.*

---

CASE V.—*Hip Joint Disease.*—Within the last ten years, great and important changes have taken place in surgical teaching and treatment respecting this disease. It was formerly supposed that patients who suffered from hip disease were scrofulous or tuberculous in constitution, and that a fatal result was generally to be looked for. You will find it said in one of the most comprehensive of our recent surgical works, that whatever inflammation is present in these cases, "we have always to bear in mind that it is of a low character, and controlled or modified by the constitutional condition; it is to the relief, therefore, of the constitutional condition that our treatment has to be mainly directed." Accordingly, Cod Liver Oil, Whiskey, and the combinations of phosphorus were the agents mainly relied on by physicians in their method of treatment. But these cases do very often recover, and in fact I do not recollect of a fatal case of simple hip-joint disease, where a rational method of treatment was employed. We see men walking the streets every day who are recoverersfrom hip-joint disease and of which the only apparent result is the slight degree of lameness it has left. The impression that hip-joint disease has any necessary connection with tuberculosis or scrofula is unfounded, as these numerous cases of recovery sufficiently attest.

The little girl who is presented before you has the characteristic features of the affection, which are flexion, together with slight adduction of the femur, with a disposition to let the muscles of the thigh remain as nearly uncontracted as possible. The weight of the body can generally be borne by the affected limb, but not without more or less pain. It is a physiological fact that pressure in the region of the hip, upon the nervous trunks supplying the knee, will cause a pain which is referred to to the knee. We should take care not to be misled on this point which forms a prominent feature in the diagnosis of these cases, and avoid the error of locating the trouble at the knee.

Hip disease occurs generally in young persons. This would not be the case were the affection tuberculous in origin. It occurs as the result, I am led to believe, of direct injury to the joint structures, such as is likely to happen to swimmers while bathing, or to one who is taking violent exercise. From the low sensibility of the joint tissues, injuries done them may not be recognized at first, while from their slow disposition to take on an inflammatory condition, changes which are taking place may pass for some time unnoticed or unheeded, till the severity of the symptoms restrain the patient from accustomed pursuits.

The diseased condition may involve the articulating extremity of either bone of the joint or its articular cartilage and synovial membrane. We find that there are two well marked stages in the progress of the affection. The first is termed acute, and is characterized by the activity and severity of the inflammation which is present. The second stage is one of less active symptoms, in which it is supposed that the processes of suppuration of injured parts and their repair, is in full progress. Abscesses may burrow their way and appear at the surface in the vicinity of the joint, through which nature endeavors to discharge the products of suppurative inflammation. The point at which these abscesses reach the surface gives very little information respecting the precise point whence they proceed, or the exact location of the diseased structure. The extent of the pathological change varies with the severity of the original injury and the care with which the suppurating structures are treated.

The treatment of diseases of the hip-joint as recommended by surgeons, to-day, is mainly local: it is *rest and relief of pressure.* By this is not meant rest from bodily activity, as those who become afflicted with this disease are oftentimes remarkably healthy and active in constitution, and it is not till the diseased condition has progressed considerably that they become emaciated and care-worn. By *rest* is meant the avoidance of active exertion of the affected extremity, and of continual motion of the diseased joint surfaces upon each other, whereby injury may be inflicted to the diseased structure, which will in the severity of its results equal that originally received, or such constant irritation and inflammation may be kept up, as will indefinitely prolong the suppurative process. In order to secure freedom from this source of injury and irritation, the patient may be kept in bed, and the affected parts be gently relieved of pressure by the keeping up of a sufficient degree of extension upon the limb, by means of a weight over a pulley. In this way the conditions of the treatment will be admirably fulfilled: the diseased surfaces relieved of pressure will cause the patient comparatively little pain, while circumstances favorable to the recovery of the affected parts will thus be obtained. It is not well to keep up very great force of extension upon the muscles of the thigh, as this may tend to their irritation and spasmodic contractraction, but simple enough to relieve that tonic contractile force which would otherwise have to be sustained by the sensitive and disorganized joint structures. Though this means is very effectual, and may be employed for a limited period, still the close confinement, to which the patient is quite unaccustomed, will soon have a very depressing effect upon his general health upon which the suppurative process is also of course making a great demand. It is then that tuberculosis, perhaps, may appear, and that tonic remedies are of use.

Happily we can secure rest of the limb and relief of pressure upon the diseased surfaces by making use of the extension splints, which have recently been introduced. They are all similar in construction to the instrument I now show you. It consists of simply a firm rod, of sufficient length to reach from the crest of the ilium nearly to the outer malleolus, to the upper portion of which is buckled a firm elastic strap, which is passed underneath the perineum, and constitutes the point of counter-extension, while to its lower extremity is buckled the ends of adhesive straps, which are wrapped spirally upon the leg, from the knee downwards. When this is applied the patient may walk about on crutches. By this excellent instrument we gain exactly what is secured by keeping the patient in bed, while the ill effects of close confinement are altogether avoided. These instruments, simple in construction, are of immense advantage, and experience has proved their worth.

Notwithstanding the value and importance of correct information concerning this affection, surgical authors have so far neglected mention of what seems to me the only rational method of treatment. Even in the most recently revised work on surgery, to which my attention has been called, I find leeching, cupping, and caustic issues unhesitatingly enjoined in the treatment of this disease, and this in conjunction with the internal administration of mercury with chalk, the mild chloride of mercury, or the bichloride, with sarsaparilla and bark. Such advice is plainly at variance with pathological fact, is for the most part useless, still further, is injurious, painful, and, in short, barbarously absurd.

If collections of pus can be found in these cases, where they can be evacuated, they should be opened. Should you have a case in which the whole head of the femur becomes carious, it may be exsected, and I have specimens to show you of this bone thus removed, whose removal was followed by recovery.

CASE VI.—*Resection of Bone after Amputation.*—The young man whose right arm you notice has been amputated in its upper third, now has protrusion of the end of the humerus, through the surrounding integument. The cause of this unfortunate result may be due to there having been an insufficiency of flaps afforded at the amputation, so that on account of the increased tension the tissues were afterwards called upon to bear, they were forced down about the bone; or the extremity of the bone left may have been so sharp edged as to cut its way out. In making amputations it is of course necessary not only to have sufficient tissue left to cover the bone, but to make allowance for the tension which subsequent inflammation will cause, when the closing stitches are liable to be torn out, and such pressure of the tissues upon the end of the bone used as will occasion such a result as is here seen. Another important fact to be regarded is that in sawing of the bone great care should be taken not to detach the periosteum from that portion of the bone which is to be left, as otherwise exfoliation is the consequence. It is also quite necessary to ream off the sharp circumference of bone which the saw leaves, as may be done by means of sharp bone forceps. Great importance is to be attached to this point, and the careful surgeon will not neglect to take the necessary pains it involves. I have found by experience that efforts in the careful removal of all acutely projecting points or edges, of bone, are amply rewarded. Though nature ultimately rounds off the sharp corners, still it is much better to assist the process yourself, and at once be rid of one great cause of delayed cicatrization and of tender stumps. In the present case, section of the cicatrix is made, a small portion of exfoliated bone taken out, and the unnecessary portion of the humerus removed by the chain saw.

